# Influence of dietary intervention on microvascular endothelial function in coronary patients and atherothrombotic risk of recurrence

**DOI:** 10.1038/s41598-021-99514-3

**Published:** 2021-10-13

**Authors:** Marta Millan-Orge, Jose D. Torres-Peña, Antonio Arenas-Larriva, Gracia M. Quintana-Navarro, Patricia Peña-Orihuela, Juan F. Alcala-Diaz, Raul M. Luque, Fernando Rodriguez-Cantalejo, Niki Katsiki, Jose Lopez-Miranda, Pablo Perez-Martinez, Javier Delgado-Lista

**Affiliations:** 1 Lipids and Atherosclerosis Unit, Internal Medicine Unit, Reina Sofia University Hospital, 14004 Cordoba, Spain; 2 Maimonides Biomedical Research Institute of Cordoba (IMIBIC), 14004 Cordoba, Spain; 3 Department of Medical and Surgical Sciences, University of Cordoba, 14004 Cordoba, Spain; 4 CIBER Fisiopatologia de La Obesidad y Nutricion (CIBEROBN), Instituto de Salud Carlos III, 28029 Madrid, Spain; 5 Department of Cell Biology, Physiology, and Immunology, University of Cordoba, Agrifood Campus of Internal Excellence (ceiA3), 14071 Cordoba, Spain; 6 Biochemical Laboratory, Reina Sofia University Hospital, Córdoba, Spain; 7 First Department of Internal Medicine, Diabetes Center, Division of Endocrinology and Metabolism, AHEPA University Hospital, 1st Stilponos Kyriakidi, 546 21 Thessaloniki, Greece

**Keywords:** Medical research, Translational research

## Abstract

Endothelial dysfunction is a key player in both the onset and development of atherosclerosis. No study has examined whether healthy dietary patterns can improve microvascular endothelial function in patients with coronary heart disease (CHD) in the long-term and whether this relationship can affect patient’s risk of CHD recurrence. In the CORDIOPREV study, a randomized, double-blind, controlled trial, dietary intervention with either the Mediterranean diet or a low-fat diet was implemented in 1,002 CHD patients. A laser-doppler flowmetry was performed at baseline and after 6 years of follow up in 664 patients, evaluating the effects of this dietary intervention on microvascular basal flow and reactive hyperaemia area, as well as on the risk of CHD recurrence, based on the TRS2P risk score. Basal flow (97.78 ± 2.79 vs. 179.31 ± 5.06 arbitrary perfusion units, 83.38% increase, *p* < 0.001) and reactive hyperaemia area (4233.3 ± 127.73 vs. 9695.9 ± 205.23 arbitrary perfusion units per time, 129.04% increase, *p* < 0.001) improved after the dietary intervention in the cohort, without finding differences due to the diet (*p* > 0.05 for the diet-effect). When patients were stratified to low, moderate or high-risk of recurrence, basal flow was similarly increased in all three groups. However, reactive hyperaemia area was improved to a greater extent in patients at the low-risk group compared with those at moderate or high-risk. No differences were observed between diets. Healthy dietary patterns can improve microvascular endothelial function and this improvement persists in the long-term. Patients with a low-risk of CHD recurrence show a greater improvement in reactive vasodilation to ischemia than patients in the moderate or high-risk groups.

## Introduction

Endothelial dysfunction is one of the key processes in the development and progression of atherosclerosis^1^. In this context, the endothelium malfunctions mainly due to a lack of bioavailability of nitric oxide (NO), which, in turn, encourages platelet adhesion and aggregation, smooth cell proliferation and migration of leukocytes in the vascular bed, leading to the establishment of a prooxidant, proinflammatory and procoagulant environment^2^. Furthermore, common comorbidities of atherosclerosis, including hypertension, diabetes mellitus, obesity and dyslipidemia enhance the production of reactive oxygen species, thus promoting atherothrombosis^1^. Therefore, endothelial dysfunction is currently identified as a key contributor to atherogenesis and the development of atherosclerotic plaque^1,2^.

Endothelial function (EF) can be evaluated at different blood vessel levels, i.e. at large, medium and microvascular sites. Different methodologies have been developed specifically for each site, as vascular biology in the different types of vessels responds differently to stimuli^3^. Several cardiovascular risk factors have been linked to an impaired EF^4^. Consumption of a healthy dietary pattern can modulate EF^5–7^. Indeed, previous studies have shown that the consumption of a Mediterranean diet (MedDiet), rich in extra virgin olive, improved EF in patients with obesity, hypercholesterolaemia, diabetes mellitus or metabolic syndrome in the short term^5,8,9^. In the CORDIOPREV study, we have previously found a favorable effect of the MedDiet on EF in the brachial artery, measured by flow-mediated dilation (FMD), in patients with coronary heart disease (CHD)^10,11^. This improvement in FMD correlated with a reduction in the concentration of endothelial microparticles and the intracellular production of reactive oxygen species, as well as an increase in endothelial progenitor cells^11^. Other healthy dietary patterns, such as a low-fat diet (LFD), have yielded divergent results in terms of their effect on EF^7,12–15^. Of note, among the different vascular beds used to evaluate EF, the brachial artery EF (assessed by FMD) has been the most studied. In this technique, echography of the brachial artery, before and after cuff-induced hypoxia, reveals the reactivity of medium-sized vessels^16^.

The microvascular system constitutes a special vascular site in human biology. The capillaries have certain structural characteristics that affect the regulation of their function, mainly their vasodilatory capacity, with circulating substances playing an important role in this process^17^. Microvascular EF (MEF) has been identified as a major homeostatic regulator, since most of the oxygen delivery to cells is carried out by blood capillaries^17^. The best-established method to evaluate MEF is laser-doppler flowmetry (LDF), an easy, fast, semi-automatic method to assess blood flow in capillaries in a similar way to pulsioxymetry^18–21^. The main factors usually assessed by LDF are: (1) basal flow (BF), i.e. the amount of blood flow that passes through the capillaries in a resting condition, and, (2) the reactive hyperaemia area (RHA), which expresses the capacity of the microvascular endothelium to vasodilate in the presence of an acute ischemia^22^.

MEF has been positively associated with the concentrations of phenol compounds^23^, NO^24^ and endothelial progenitor cells^25^. In this context, consumption of a MedDiet for 4 weeks increased the circulating levels of these substances and improved MEF^23–25^. However, up-to-date, there are no studies evaluating the long-term impact of healthy diets on the MEF in CHD patients, or whether this impact is linked to their risk of CHD recurrence. This risk may be assessed by the “Thrombolysis in Myocardial Infarction Risk (TIMI) Score for Secondary Prevention” (TRS2P), a tool that can categorize the risk of recurrence in patients with atherosclerotic cardiovascular disease (ASCVD)^26^.

The aim of the current study was to evaluate whether the consumption of healthy diets (MedDiet or LFD) influences the MEF of CHD patients in the long-term, and whether this influence may interrelate with the risk of CHD recurrence, assessed by the TRS2P score.

## Patients and methods

### Overall design and patient population

The CORDIOPREV study (Clinicaltrials.gov number NCT00924937) is a randomized, double-blind, controlled trial including 1,002 CHD patients. The study was conducted at the Instituto Maimonides de Investigacion Biomedica de Cordoba (IMIBIC), a scientific institute which carries out research into biomedical areas for the Reina Sofia University Hospital, and the University of Cordoba, Spain. The lipids and atherosclerosis unit, internal medicine unit, is also a member of the CIBER Fisiopatologia de la Obesidad y Nutrición (CIBERobn), a national research organization studying obesity and nutrition and their impact on health and disease.

### Objectives and interventions

The aim of the CORDIOPREV study was to examine the efficacy of a MedDiet rich in olive oil, compared with a LFD, to prevent new, major cardiovascular clinical events. The baseline characteristics, inclusion and exclusion criteria have been published previously^27^ (See the Supplemental Materials). The aim of the current study was to evaluate whether the consumption of these two healthy diets influences the MEF of CHD patients in the long-term, and whether this influence may interrelate with the risk of CHD recurrence, assessed by the TRS2P. MEF was measured at baseline and during follow-up. The CORDIOPREV study protocol was approved by the local ethics committee, in line with the Helsinki declaration and good clinical practices, and all patients gave their informed consent to participate in the study.

The current study included only those patients who were active, following the study protocol and who had an LDF performed at 6 years of follow-up.

### Assesment of MEF

LDF (Periflux 5000, Perimed SA) was used to assess MEF at baseline and after 6 years. LDF evaluates cutaneous microcirculation in real time through a laser probe situated on the second finger of the dominant hand^22^. This technique also assesses ischemia-induced effect in a specific site, as a stimulus for microvascular endothelial vasodilation. Ischemia is induced by inflating a blood pressure cuff over suprasystolic pressure (200 to 220 mmHg) for 4 min in the upper arm, 2–4 cm above the elbow bend. BF and RHA were measured. BF was expressed as arbitrary perfusion units (APU) and RHA as arbitrary perfusion units per time (APUT)^22^.

### Patient classification based on their baseline risk of CHD recurrence as assessed by the TRS2P risk score

**Figure 1:**
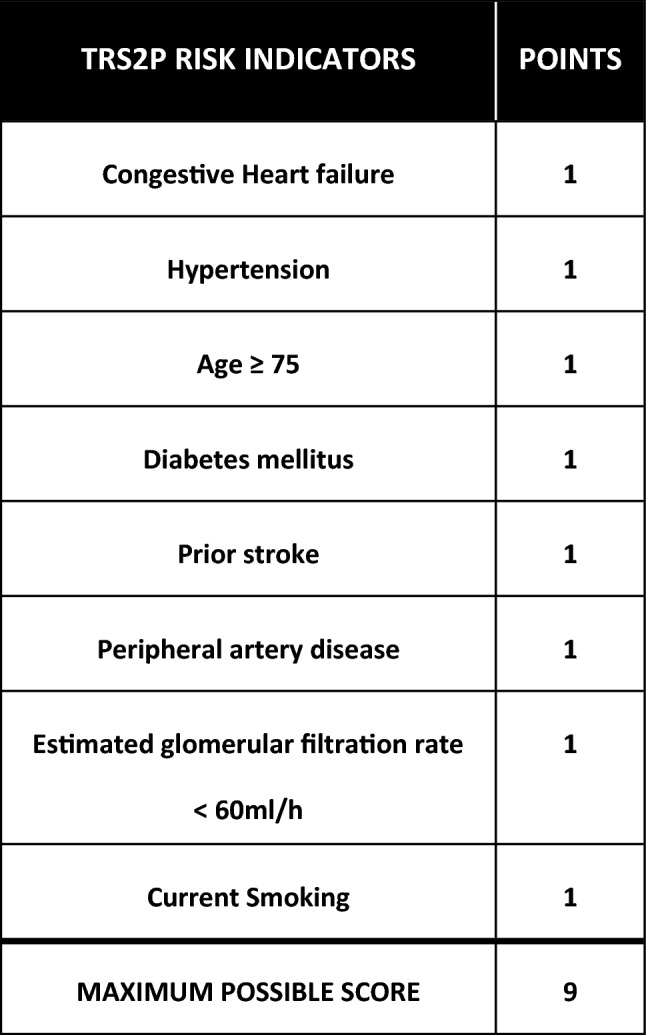
TRS2P risk indicators. Self-production. Source: Bohula et al.^26^.

To assess whether the effect of dietary intervention on the MEF of CHD patients can be related to their risk of CHD recurrence, patients were classified according to the TRS2P score. This tool is used to predict the risk of recurrence in several populations, and is based on 9 clinical entities identified as independent factors of new atherothrombotic events (Fig. 1). Each item represents 1 point. Total score is the sum of all clinical comorbities present in each individual. According to previous findings^28–30^, patients were classified into three groups: low (0–1 points), moderate (2 points) and high atherothrombotic risk (> 2 points).

### Statistical analysis

Continuous variables are expressed as mean ± standard error,whereas percentages were used to express dichotomous variables. Long-term changes in BF and RHA at 6 years were assessed with a T-test for related samples. Repeated-measures ANOVA and post-hoc multiple comparisons analysis with Bonferroni’s correction were used to investigate the effects of diets on BF and RHA and the differences between atherothrombotic risk groups. The chi-square and the Monte Carlo test were performed to evaluate the distribution of the TRS2P components among the three risk groups. All analyses were adjusted for gender and drug therapy (statins, beta-blockers, angiotensin-converting enzyme inhibitors and angiotensin receptor blockers) at baseline. A 2-sided *p* value < 0.05 was considered statistically significant. All statistical analyses were performed by the use of SPSS 23 (IBM SPSS Statistics for Windows, Version 23.0. Armonk, NY: IBM Corp).

### Ethics approval and consent to participate

All the patients gave their written informed consent to participate in the study. Following institutional and Good Clinical Practice guidelines, the Human Investigation Review Committee (the local ethics committee “Comité de Ética de la Investigación de Córdoba (CEIC)”) approved the study protocol at Reina Sofia University Hospital.

### Consent for publication

Not applicable.

## Results

**Figure 2:**
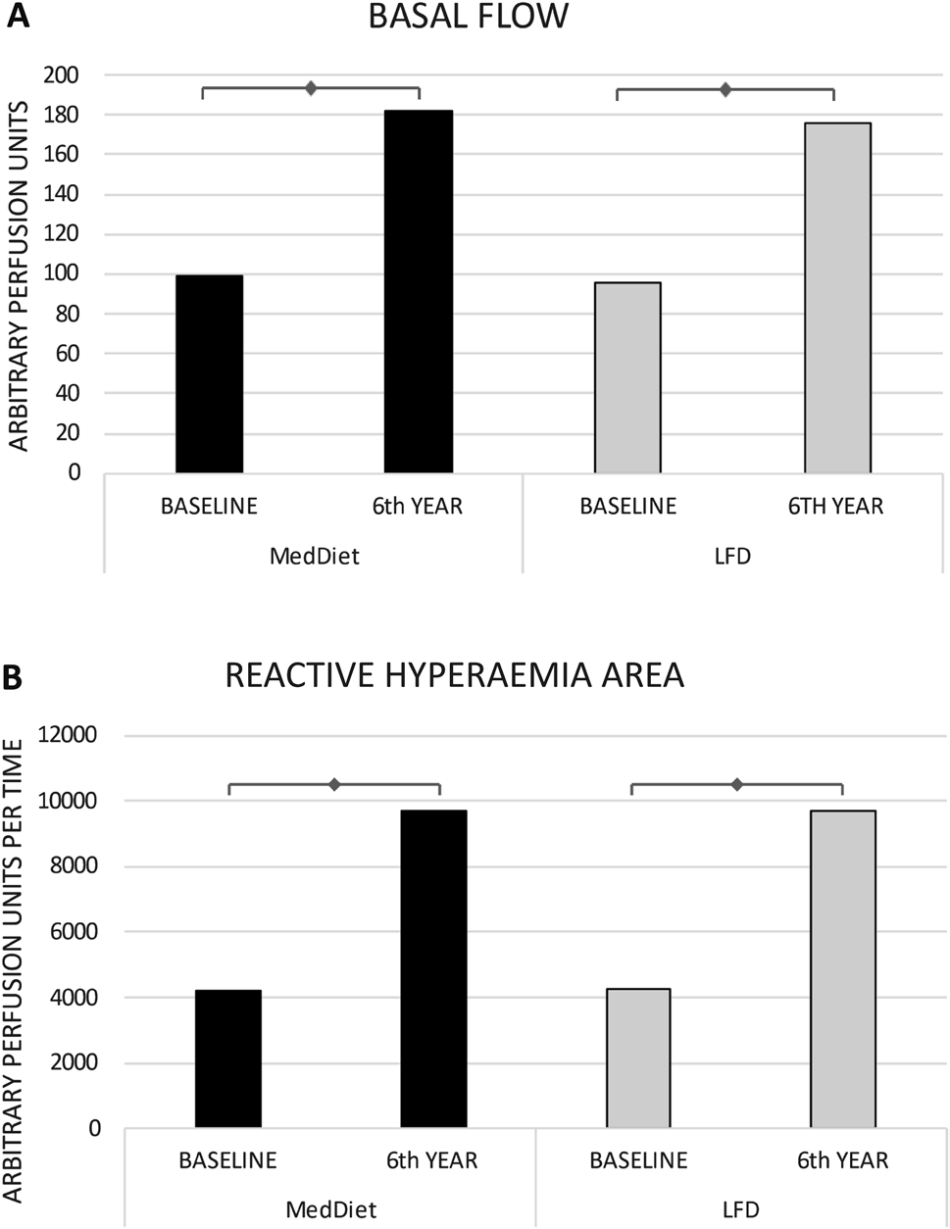
LASER DOPPLER FLOWMETRY (LDF). (**A**) Basal flow at baseline and after 6 years of consumption of MedDiet or LFD. (**B**) Reactive hyperaemia area at baseline and after 6 years of consumption of MedDiet or LFD. MedDiet, mediterranean diet; LFD, low fat diet. ◊ indicates significant differences (*p* < 0.05).

From the initial 1,002 CHD patients included in the CORDIOPREV study, a total of 664 patients were included in the study (See flow-chart included in the Supplemental Materials). Dietary intervention with either MedDiet or LFD improved both BF (97.78 ± 2.79 APU vs. 179.31 ± 5.06 APU, 83.38% increase, *p* < 0.001) and RHA (4233.3 ± 127.73 APUT vs. 9695.9 ± 205.23 APUT, 129.04% increase, *p* < 0.001). No differences were observed between the two diets (*p* = 0.69) (Fig. 2).

According to the TRS2P score, 298 patients were classified as low-risk, 243 as moderate-risk and 123 as high-risk. The clinical characteristics and TRS2P factor distribution for each risk group are shown in Table 1. At baseline, there were no differences in BF and RHA between risk groups and according to the dietary pattern (BF: 97.7 ± 6.3 vs. 87.7 ± 5.3 APU for MedDiet vs. LFD in the low-risk group; 100.4 ± 7.2 vs. 103.5 ± 6.1 APU for MedDiet vs. LFD in the moderate-risk group; 101.5 ± 8.1 vs. 98.9 ± 8.5 APU for MedDiet vs. LFD in the high-risk group, *p* > 0.05 all comparisons; RHA: 4251.96 ± 262.3 vs.4564.88 ± 300.3 APUT for MedDiet vs. LFD in the low-risk group; 4263.6 ± 256.7 vs. 3988.83 ± 306.7 APUT for MedDiet vs. LFD in the moderate-risk group; 3984.5 ± 389.5 vs. 4192.8 ± 470.8 APUT for MedDiet vs. LFD in the high-risk group, *p* > 0.05 all comparisons).

**Table 1:** Baseline characteristics of the patients, according to TRS2P risk group.

	Low risk (TRS2P < 2) n = 298	Moderate risk (TRS2P = 2) n = 243	High risk (TRS2P ≥ 3) n = 123	*p* value
Diet (% MedDiet)	55.9	48.4	56.5	0.16
Age ± SE (years)	57.4 ± 0.5^a^	60.5 ± 0.5^b^	61.7 ± 0.8^b^	< 0.001
Sex (% men)	82.6	81.1	89.5	0.11
**Cardiovascular risk and TRS2P factors**				
Age ≥ 75 years (%)	0.7^a^	2.5^a^	8.9^b^	< 0.001
Smokers (%)	7.4^a^	18.9^b^	44.7^c^	< 0.001
DM2 (%)	21.8^a^	77^b^	92.7^c^	< 0.001
HTA (%)	46.6^a^	82.3^b^	92.7^c^	< 0.001
MI (%)	66.8^a^	56^b^	56.1^b^	0.018
Stroke (%)	1^a^	4.9^b^	14.6^c^	< 0.001
PAD (%)	0^a^	4.9^b^	25.2^c^	< 0.001
Coronary Bypass (%)	0^a^	3.7^b^	9.8^c^	< 0.001
Heart failure (%)	0.7^a^	2.5^a^	12.2^b^	< 0.001
Renal failure (%)	0.3^a^	3.3^b^	23.6^c^	< 0.001

TRS2P, Thrombolysis in Myocardial Infarction Risk (TIMI) Score for Secondary Prevention; MedDiet, mediterranean diet; DM2, diabetes mellitus type 2; HTA, hypertension; MI, myocardial infarction; PAD, peripheral artery disease; SE, standard error.

Values in the same row with different letters are significantly different.

**Figure 3:**
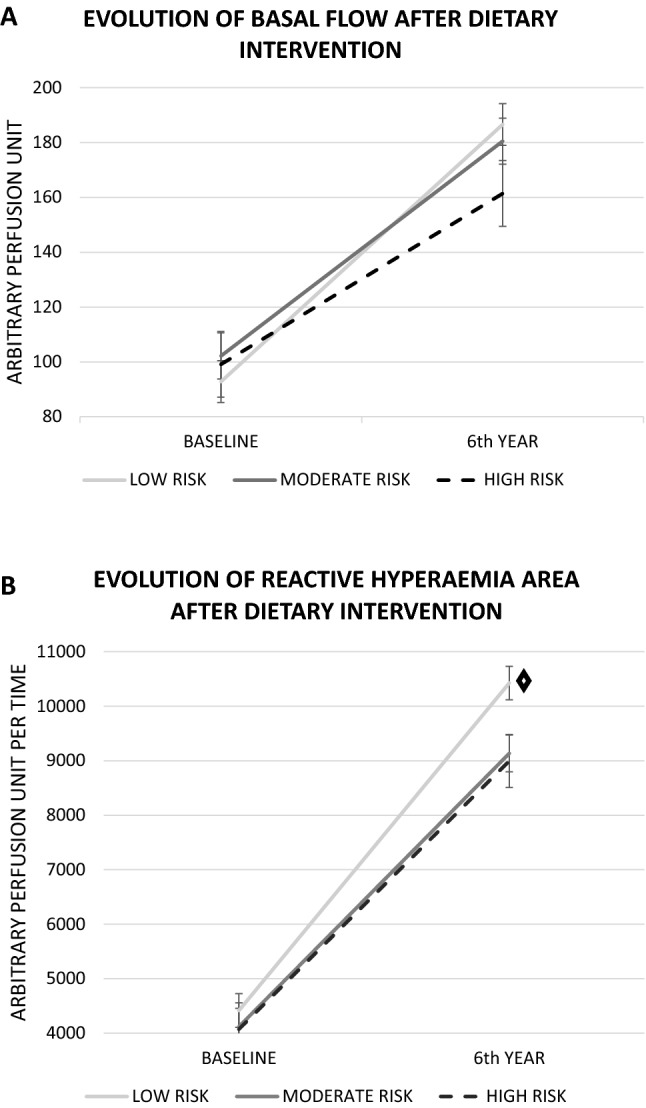
LASER DOPPLER FLOWMETRY (LDF). (**A**) Basal flow at baseline and after 6 years according to TRS2P risk groups. (**B**) Hyperaemia area at baseline and after 6 years according to TRS2P risk groups. ◊ indicates significant differences between low-risk versus moderate- and high-risk groups (*p* < 0.05).

BF and RHA significantly improved in the total population after 6 years of follow-up in all risk groups (BF: 92.83 ± 4.2 vs. 186.62 ± 7.6 APU in the low-risk group; 102.2 ± 4.6 vs. 180.5 ± 8.4 APU in the moderate-risk group; 99.12 ± 6.6 vs. 161.46 ± 12 APU in the high-risk group; RHA: 4416.6 ± 193.2 vs. 10,424.59 ± 308.5 APUT in the low-risk group; 4120.28 ± 211.9 vs. 9133.16 ± 338.3 APUT in the moderate-risk group; 4073.02 ± 303.7 vs. 8995.58 ± 484.82 APUT in the high-risk group; *p* < 0.001 all comparisons) (Fig. 3). We didn´t find any effect of diet in these changes(Supplemental Material).

BF improvement was similar between the three atherothrombotic risk groups (*p* = 0.47), but RHA was greatly improved in the low-risk group compared with both the moderate and high-risk groups (*p* < 0.05) (Fig. 3).

## Discussion

The current study found that the consumption a healthy dietary pattern (MedDiet or LFD) over 6 years can lead to a long-term improvement in MEF in patients with CHD. Indeed, both BF and RHA improved compared with baseline measurements, regardless of the type of healthy diet. Furthermore, although this benefit was evident in all patients, those at low-risk of CHD recurrence, according to the TRS2P score, showed an even greater improvement in their RHA, compared with the moderate and high-risk groups.

EF is impaired in patients with hypertension, diabetes mellitus and hypercholesterolemia^17,31–33^. Drug treatment of these conditions was shown to improve macro- and microvascular EF in the short-term^3,31–36^. On the other hand, different dietary patterns may modulate EF. In a previous report from our group, the long-term consumption of a MedDiet, rich in extra virgin olive oil, for 1.5 years improved FMD in the brachial artery in patients with diabetes and prediabetes, while the consumption of a LFD stabilized FMD^10^. A recent meta-analysis, including 7 randomized controlled trials using FMD, confirmed these results^37^. Regarding LFD, the evidence on its impact on EF is controversial with some authors reporting an improvement in EF after a few weeks of LFD consumption^7,13^, and others finding that EF remained stable after one year of follow-up^12,15^. Of note, the available studies focused on assessing the effect of LFD in obese or diabetic patients without ASCVD^7,12–15^. Up-to-date, it had not been clarified whether these effects of healthy diets on EF, especially in MEF, persisted or disappeared over time.

With regard to microcirculation, previous studies demonstrated that MedDiet increased MEF at 8 weeks in patients over 65 years, alone or in combination with exercise ^25,38^. This benefit was also observed in patients with hypercholesterolemia or metabolic syndrome, when evaluating postprandial MEF with LDF ^23,24^. There are varying underlying mechanisms that may account for the benefits of a MedDiet on MEF. On one hand, extra virgin olive oil is rich in monounsaturated fatty acids (MUFAs) and phenolic compounds, both of which reduce superoxide production and decrease the oxidation of low-density lipoprotein (LDL) particles, thus inhibiting the development of atherosclerotic plaque and endothelial dysfunction^39,40^. On the other hand, oily fish increases NO bioavailability, by upregulation of endothelial NO synthase, whereas green vegetables, grains and legumes are the provisions of inorganic nitrate and L-arginine, which are NO precursors^37^. Oxidative stress, inflammation and endothelial dysfunction are interrelated^1^. In fact, long-term consumption of a MedDiet rich in extra virgin olive oil was linked to a reduction in the plasma/serum concentration of pro-inflammatory markers such as interleukin-6, a C-reactive protein, adhesion molecules and in the chemokines in the PREDIMED (Prevención con Dieta Mediterránea) Study^41,42^, where the MedDiet reduced the relative risk of major cardiovascular events in patients without ASCVD. Most of the evidence about LFD derives from studies invovling vascular beds other than microcirculation. Therefore, the findings of the current study are the first to assess long-term impact of LFD on microcirculation with laser-doppler technology, and confirmed that both MedDiet and LFD can improve MEF in patients with CHD.

In the current study, patients were classified according to TRS2P to evaluate whether long-term changes in MEF after the implementation of a healthy dietary pattern were associated with individual’s risk of CHD recurrence. This hypothesis could help to implement diverse dietary approaches for patients with a different CHD severity, with a more personalized approach. We used the TRS2P score since this tool was also applied in the *Examining Outcomes in Subjects With Acute Coronary Syndrome: Vytorin (Ezetimibe/Simvastatin) vs Simvastatin* (IMPROVE-IT)^28^, *The Saxagliptin assessment of vascular outcomes recorded in patients with diabetes mellitus-thrombolysis in myocardial infarction* (SAVOR-TIMI)^30^ or *the Further Cardiovascular Outcomes Research With PCSK9 Inhibition in Subjects With Elevated Risk* (FOURIER)^29^ clinical trials which all evaluated the efficacy of pharmacological therapies to decrease atherothrombotic events and mortality in patients with established ASCVD or very high cardiovascular risk. The results from these clinical trials showed that patients classified as low-risk had a lower incidence of atherothrombotic events than the high-risk group in the following 24–36 months.

The aim of the current sub-study from the CORDIOPREV trial was not to evaluate the incidence of atherothrombotic events, but to compare the changes in MEF between risk categories according to TRS2P in the environment of a long-term dietary intervention. We found that both MedDiet and LFD improved MEF in a similar way in the low, moderate and high atherothrombotic risk groups. However, the low-risk TRS2P group showed a greater improvement in microvascular reactivity (assessed by the RHA) compared with the moderate and high-risk groups. These findings suggest that, although a healthy dietary pattern can beneficially affect BF in all patients in a similar way, microvascular reactivity to ischemia improves more in low-risk patients according to TRS2P. In previous studies, only patients classified as moderate or high-risk benefited from more intensive pharmacological treatment ^28–30^. However, in the current analysis, it seems that the low-risk patients benefited most from the dietary intervention. Therefore, although this improvement in MEF has not been correlated with a lower incidence of cardiovascular events yet, the role of dietary recommendations in all patients with cardiovascular disease should be highlighted, especially in low-risk groups who may not benefit from more intensive drug therapy and who may gain more from adherence to a healthy dietary pattern, according to the findings of the current study.

To our knowledge, this is the largest study up-to-date evaluating the long-term effects of two dietary healthy patterns on MEF (i.e., BF and RHA) in patients with CHD. However, there are certain limitations, including the lack of possibility of generalization to other populations without CVD, the fact that Mediterranean Diet was used in a country where it has a high acceptation and may not be extrapolated to other countries, or that we performed a high intensity dietary intervention which could be difficult to achieve in other settings.

In conclusion, a long-term healthy dietary intervention with MedDiet or LFD significantly improved microvascular BF in CHD patients, regardless of their baseline atherothrombotic risk (defined by the TRS2P). Microvascular reactivity to ischemia (as assessed by RHA) also improved in all CHD patients following a healthy dietary pattern, but this improvement was greater in patients at the low-risk category compared with those at the moderate and high-risk groups. Further research is needed to establish the long-term benefits of dietary intervensions in MEF in different patient populations.

## Supplementary Information

Supplementary Information.

## Data Availability

Data supporting the findings of this study may be requested to  the corresponding author. Propierty of Data Policy from the Cordioprev Study apply to this article.

## Abbreviations

CHD: Coronary heart disease
NO: Nitric oxide
EF: Endothelial function
MedDiet: Mediterranean diet
FMD: Flow-mediated dilation
LFD: Low-fat diet
MEF: Microvascular EF
LDF: Laser-doppler flowmetry
BF: Basal flow
RHA: Reactive hyperaemia area
TIMI: Thrombolysis in Myocardial Infarction Risk
TRS2P: TIMI Score for Secondary Prevention
ASCVD: Atherosclerotic cardiovascular disease
APUT: Arbitrary perfusion units per time
MUFAs: Monounsaturated fatty acids
LDL: Low-density lipoprotein
