# Supplementary material for: Influence of dietary intervention on microvascular endothelial function in coronary patients and atherothrombotic risk of recurrence
Source: Sci Rep. 2021 Oct 13;11:20301. doi: 10.1038/s41598-021-99514-3 (PMC8514425; doi:10.1038/s41598-021-99514-3)
Supplement: Supplementary file 1 — Supplementary Information. [file 41598_2021_99514_MOESM1_ESM.docx]

**SUPPLEMENTAL MATERIAL**

|  | **TOTAL**  **(n=664)** | | | | | | **MedDiet group**  **(n=355)** | | | | | | **LFD**  **(n=309)** | | | | | | **p value*** |
| --- | --- | --- | --- | --- | --- | --- | --- | --- | --- | --- | --- | --- | --- | --- | --- | --- | --- | --- | --- |
|  | LOW RISK | | MODERATE RISK | | HIGH RISK | | LOW RISK | | MODERATE RISK | | HIGH RISK | | LOW RISK | | MODERATE RISK | | HIGH RISK | |  |
|  | **BASELINE** | **6^TH^ YEAR** | **BASELINE** | **6^TH^ YEAR** | **BASELINE** | **6^TH^ YEAR** | **BASELINE** | **6^TH^ YEAR** | **BASELINE** | **6^TH^ YEAR** | **BASELINE** | **6^TH^ YEAR** | **BASELINE** | **6^TH^ YEAR** | **BASELINE** | **6^TH^ YEAR** | **BASELINE** | **6^TH^ YEAR** |  |
| **Basal flow ± SE (APU)** | 92.83 ± 4.2 | 186.62 ± 7.6 | 102.2 ± 4.6 | 180.5 ± 8.4 | 99.12 ± 6.6 | 161.46 ± 12 | 98.32 ± 5.6 | 187.21 ± 10.13 | 101.33 ± 6.6 | 192.51 ± 12.05 | 101.14 ± 8.7 | 149.55 ± 15.76 | 87.34 ± 6.33 | 186.02 ± 11.47 | 103.07 ± 6.45 | 168.49 ± 11.68 | 97.11 ± 9.93 | 173.36 ± 18.04 | 0.73 |
| **Reactive hyperemia area ± SE (APUT)** | 4416.6 ± 193.2 | 10424.59 ± 308.5 | 4120.28 ± 211.9 | 9133.16 ± 338.3 | 4073.02 ± 303.7 | 8995.58 ± 484.82 | 4282.85 ± 256.06 | 10477.77 ± 408.82 | 4257.04 ± 304.78 | 8896.72 ± 486.59 | 3974.86 ± 398.51 | 9123.12 ± 636.25 | 4550.37 ± 289.9 | 10372.4 ± 462.86 | 3983.51 ± 295.35 | 9369.6 ± 471.54 | 4171.19 ± 455.18 | 8868.03 ± 726.73 | 0.85 |

**Supplemental Table 1. Evolution of microvascular endothelial function according to diet group.** APU, arbitrary perfusion units; APUT, arbitrary perfusion units per time; LFD, low fat diet; MedDiet, Mediterranean diet; SE, standard error. *****The p-value corresponds to the effect of the diet.

**Supplemental Figure 1. Flowchart of the study subjects.**

**Supplemental table 2**

Inclusion and exclusion criteria for CORDIOPREV study.

| **Inclusion Criteria in the CORDIOPREV study:** |
| --- |
| 1.- Informed Consent: All participants will agree to being included in the study by signing the protocol approved by the Reina Sofia University Hospital Clinical Research Ethics Committee. In this written statement of consent, it will state that patients will be chosen for inclusion in the groups on a random basis.  2.- Diagnostic Criteria: The patients were selected with acute coronary syndrome (unstable angina, acute myocardial infarction) and high-risk chronic coronary heart disease, according to the following criteria:   1. Acute myocardial infarction: The existence of at least two of the following three signs: angina-type chest pain (or anginal equivalents), typical ECG changes (appearance of new Q waves and/or changes in ST segments and/or T waves), and a rise in myocardial enzymes (CPK and/or CPK/MB> of twice the normal laboratory limits). The MB value criterion will prevail in case of discrepancies over the total CPK. 2. Unstable angina: Admission to hospital for angina-type chest pains lasting at least 15 minutes, both at rest and after exercise, which have increased in frequency and duration in recent days or weeks. The latest episode must have occurred at least 48 hours prior to admission and must be accompanied by at least one of the following electrocardiographic or analytical changes:    - ST depression of at least 0.5 mm in 2 contiguous leads.    - ST elevation of at least 1 mm in 2 contiguous leads.    - T-wave inversion of at least 2 mm in 2 contiguous leads.    - Positive troponin result. 3. Chronic high-risk ischemic heart disease: patients will be included who have been hospitalized for a coronary event and/or stable angina, at least once in the past 2 years and who have undergone diagnostic coronary angiography with evidence of severe coronary disease, which is defined as the existence of an epicardial vessel greater than 2.5 mm in diameter with stenosis of over 50%. |
| **Exclusion Criteria** |
| 1. Patients under 20 years of age or over 75 years old with a life expectancy of over 5 years. 2. Severe heart failure, NYHA functional class III or IV, with the exception of self-limited episodes of acute heart failure at the time of the acute ischemic event. 3. Severe left ventricular systolic dysfunction (with ejection fraction equal to or under 35%). 4. Patients with restricted capacity to follow the protocol: those unable to follow the prescribed diet for whatever reason, due to personal or family circumstances. 5. Risk factors which are severe or difficult to control: patients with hypertension and diabetes, where there is organ involvement that limits their survival, will be excluded (chronic renal failure with creatinine which is persistently >2.5 mg/dl) and disabling clinical manifestations of cerebral atherosclerosis. 6. Chronic diseases unrelated to coronary risk: severe psychiatric illnesses, chronic conditions requiring treatment that could modify the lipid metabolism (chronic renal failure, chronic liver disease, neoplasia under treatment, chronic obstructive pulmonary disease involving respiratory pulmonary failure with home oxygen therapy, endocrine diseases susceptible to decompensation and diseases of the digestive tract that involve episodes of diarrhea). 7. Participants in other studies: patients taking part in other studies will be excluded, at the time of selection or up to 30 days before the study begins. |

Information from: doi:10.1016/j.ahj.2016.04.011
